# The Ability of Metabolomics to Discriminate Non-Small-Cell Lung Cancer Subtypes Depends on the Stage of the Disease and the Type of Material Studied

**DOI:** 10.3390/cancers13133314

**Published:** 2021-07-01

**Authors:** Tomasz Kowalczyk, Joanna Kisluk, Karolina Pietrowska, Joanna Godzien, Miroslaw Kozlowski, Joanna Reszeć, Ewa Sierko, Wojciech Naumnik, Robert Mróz, Marcin Moniuszko, Adam Kretowski, Jacek Niklinski, Michal Ciborowski

**Affiliations:** 1 Metabolomics Laboratory, Clinical Research Centre, Medical University of Bialystok, M. Skłodowskiej-Curie 24a, 15-276 Bialystok, Poland; tomasz.kowalczyk@umb.edu.pl (T.K.); karolina.pietrowska@umb.edu.pl (K.P.); joanna.godzien@umb.edu.pl (J.G.); adamkretowski@wp.pl (A.K.); 2 Department of Clinical Molecular Biology, Medical University of Bialystok, Waszyngtona 13, 15-269 Bialystok, Poland; joanna.kisluk@umb.edu.pl (J.K.); jacek.niklinski@umb.edu.pl (J.N.); 3 Department of Thoracic Surgery, Medical University of Bialystok, M. Skłodowskiej-Curie 24a, 15-276 Bialystok, Poland; miroslaw.kozlowski@umb.edu.pl; 4 Department of Medical Patomorphology, Medical University of Bialystok, Waszyngtona 13, 15-269 Bialystok, Poland; joannareszec@gmail.com; 5 Department of Oncology, Medical University of Bialystok, Ogrodowa 12, 15-027 Bialystok, Poland; ewa.sierko@iq.pl; 6 1st Department of Lung Diseases and Tuberculosis, Medical University of Bialystok, Żurawia 14, 15-540 Bialystok, Poland; wojciech.naumnik@umb.edu.pl; 7 2nd Department of Lung Diseases and Tuberculosis, Medical University of Bialystok, Żurawia 14, 15-540 Bialystok, Poland; robert.mroz@umb.edu.pl; 8 Department of Allergology and Internal Medicine, Medical University of Bialystok, M. Skłodowskiej-Curie 24a, 15-276 Bialystok, Poland; marcin.moniuszko@umb.edu.pl; 9 Department of Regenerative Medicine and Immune Regulation, Medical University of Bialystok, Waszyngtona 13, 15-269 Bialystok, Poland; 10 Department of Endocrinology, Diabetology and Internal Medicine, Medical University of Bialystok, M. Skłodowskiej-Curie 24a, 15-276 Bialystok, Poland

**Keywords:** non-small-cell lung cancer, metabolomics, early stage, advanced stage

## Abstract

**Simple Summary:**

The most commonly diagnosed lung cancer is non-small-cell lung cancer (NSCLC). In contrast, the most frequent subtypes of NSCLC, accounting for 80% of cases, are ADC and SCC. Nevertheless, subtype identification is based on the diagnosis of characteristic gene mutations occurring in each subtype. The aim of the study was the metabolomics analysis of the early stage of NSCLC and the determination of new biochemical pathways differentiating the subtypes. Our studies demonstrated that there are new potential significant changes in the biochemical pathways involved in N-acylethanolamine (NAE) biosynthesis that distinguish early-stage SCC from ADC. Moreover, the analysis of the plasma of patients with COPD and NSCLC allows the exclusion of metabolites related to inflammation in the lungs and the identification of compounds characteristic of cancer. Our research indicates new pathways that have not been explored in NSCLC so far, which may have diagnostic, prognostic, and therapeutic potential.

**Abstract:**

Identification of the NSCLC subtype at an early stage is still quite sophisticated. Metabolomics analysis of tissue and plasma of NSCLC patients may indicate new, and yet unknown, metabolic pathways active in the NSCLC. Our research characterized the metabolomics profile of tissue and plasma of patients with early and advanced NSCLC stage. Samples were subjected to thorough metabolomics analyses using liquid chromatography-mass spectrometry (LC-MS) technique. Tissue and/or plasma samples from 137 NSCLC patients were analyzed. Based on the early stage tissue analysis, more than 200 metabolites differentiating adenocarcinoma (ADC) and squamous cell lung carcinoma (SCC) subtypes as well as normal tissue, were identified. Most of the identified metabolites were amino acids, fatty acids, carnitines, lysoglycerophospholipids, sphingomyelins, plasmalogens and glycerophospholipids. Moreover, metabolites related to N-acyl ethanolamine (NAE) biosynthesis, namely glycerophospho (N-acyl) ethanolamines (GP-NAE), which discriminated early-stage SCC from ADC, have also been identified. On the other hand, the analysis of plasma of chronic obstructive pulmonary disease (COPD) and NSCLC patients allowed exclusion of the metabolites related to the inflammatory state in lungs and the identification of compounds (lysoglycerophospholipids, glycerophospholipids and sphingomyelins) truly characteristic to cancer. Our results, among already known, showed novel, thus far not described, metabolites discriminating NSCLC subtypes, especially in the early stage of cancer. Moreover, the presented results also indicated the activity of new metabolic pathways in NSCLC. Further investigations on the role of NAE biosynthesis pathways in the early stage of NSCLC may reveal new prognostic and diagnostic targets.

## 1. Introduction

Lung cancer is one of the five most often diagnosed diseases in the world. Furthermore, it is the leading cause of death from cancer. The most common type of lung cancer is non-small-cell lung cancer (NSCLC), representing about 85% of all cases. The most frequent histological subtypes are adenocarcinoma (ADC), squamous cell lung carcinoma (SCC) and large cell carcinoma (LCC), of which ADC and SCC represent about 85% of all cases [1]. However, this traditional distinction is now over-simplified, since several oncogenic driver mutations have been found. Especially, that for several driver mutations (e.g., EGFR, ALK, ROS-1, KRAS, EML4-ALK mutations in ADC; NRF2 mutation in SCC or cMYC overexpression and inactivation of TP53 via mutations in both subtypes), targeted therapies have been developed [2,3]. Unfortunately, only up to 60% of ADC and up to 50–80% of SCC patients have a known oncogenic driver mutation. Moreover, the tumors inevitably develop drug resistance, and in case of some treatment options, even more than 60% of patients develop resistance to received therapy [4]. Therefore, despite the introduction of new treatment strategies, for many patients, classic histopathology-based therapy is the gold standard [5]. Considering what is above, and the fact that specific mutations are also characteristic to particular subtype (e.g., EGFR, ALK, and ROS1 mutations are highly associated with ADC subtype [6]), proper NSCLC subtyping is of great importance. Currently, non-invasive methods for NSCLC subtyping and early diagnosis are not available. Metabolomics is still a new field of science, which has the potential to discover metabolic pathways altered by particular disease and subsequently propose novel diagnostic markers and targets for the therapy. Such analyses can be performed on various types of biological material. Easy-to-obtain biological samples such as serum/plasma or urine are commonly used, but cancer tissue samples, which indicate in situ metabolic changes, can also be studied. However, obtaining a tissue sample for examination from a large set of patients is challenging [7]. So far, there have been few studies in which a metabolomics approach has been used to study lung cancer. There have been attempts to identify altered metabolites in NSCLC by monitoring different types of samples but mainly plasma and serum [8,9]. Analysis of the metabolomics profile of plasma showed a significant variation in the level of amino acids, carbohydrates, organic acids, fatty acids, lipids or acylcarnitines [10,11,12]. However, the results described are not always coherent. An example is the level of glutamate that in some studies was found increased in patients with NSCLC [11,13], but in the work presented by Hori et al., the level of this metabolite was reduced [14]. A similar situation was observed in the level of lactic acid [14,15]. Moreover, differences in the metabolomics profile may also depend on the stage of the disease. The metabolism of early-stage cancer differs significantly from the advanced stage due to tumor size and activity [11]. Although the latest scientific reports focus on a growing number of patients included [16], the most crucial aspect of treatment is a diagnosis of the disease at the earliest possible stage. Recent scientific reports increasingly indicate panels of metabolites that may help in the early diagnosis of lung cancer [8,10,11]. However, due to high blood homeostasis, it is difficult to find specific cancer markers in serum or plasma. Therefore, tissue analysis is a crucial element of research to identify potential lung cancer biomarkers. The tissue is the center of action in which all tumor proliferation reactions and the secretion of metabolites to the blood occur. To date, a limited number of studies have been published presenting the analysis of metabolic differences arising in lung cancer tissue. The first work was performed by Fan et al., who analyzed C13 glucose, labelled tissue extracts and observed increased levels of amino acids, lactate and citrate, indicating changes in the Krebs cycle and pyruvate carboxylation [17]. Similar studies were conducted by Hori et al., who analyzed tissue samples and identified metabolites indicating for disturbances in the TCA cycle [14]. Besides that, Kami et al. presented the results showing high glycolytic activity in lung cancer [18]. Finally, Farhmann et al. and Wikoff et al. studied early-stage lung cancer and identified various metabolic alterations associated with the ADC subtype [19,20].

Although plasma or serum are preferable over tissue samples for cancer diagnosis or monitoring treatment effects, knowledge gathered by analysis of tissue is vital for exploring cancer biochemistry. In this study, metabolic profiles of cancer tissue samples collected from NSCLC patients and adjacent normal lung tissue from the same patient as well as plasma samples collected from the same (if possible) NSCLC patients and the control group (composed of chronic obstructive pulmonary disease (COPD) patients) were obtained. Based on the histopathological examination, NSCLC patients were divided into three subgroups representing the following histological subtypes: ADC, SCC and LCC. Additionally, studied patients were divided into those in the early (TNM IA-IB) and advanced stage (TNM IIA-IIIA) of the disease. The aim of this study was to search for metabolites discriminating control tissue from cancer tissue and the plasma of NSCLC patients from the plasma of control group. Furthermore, identification of tissue and plasma metabolites that differentiate NSCLC subtypes was also undertaken. All of these comparisons were performed separately in early- and advanced-stage groups.

## 2. Materials and Methods

### 2.1. Patients’ Characteristics and Samples Collection

Lung tissue samples were obtained from patients undergoing surgical treatment for primary NSCLC at the Department of Thoracic Surgery of Medical University of Bialystok Clinical Hospital (Poland). In total, tissue samples from 99 NSCLC patients were included in this study. Both lung tumor tissue and adjacent control tissue without morphological changes were collected, histologically reviewed and classified. All tissue samples were frozen and stored at −80 °C until analysis. The samples were collected following the highest biobanking standards established at our university [21].

Plasma samples analyzed in this study were obtained from 72 patients with NSCLC and 20 patients with COPD, classified as a control group. For 45% of NSCLC patients enrolled for plasma metabolomics, lung tissue samples were also available and analyzed in this study. Whole blood was collected in 9 mL vacuum system tubes with K_2_EDTA as an anticoagulant. After gentle mixing, plasma was separated by centrifugation at 1300× *g* for 20 min at room temperature. Plasma fractions (0.5 mL each) were then collected in Eppendorf tubes and stored at −80 °C until analysis. In total, the study group was composed of 137 NSCLC patients. In case of patients enrolled for tissue analysis, according to TNM classification, 28 participants (14 with ADC and 14 with SCC subtype) were classified as early-stage NSCLC patients, while 71 patients (19 with ADC, 40 with SCC and 12 with LCC subtype) were classified as advanced-stage NSCLC patients. In the case of patients enrolled for plasma analysis, 39 participants (21 with ADC and 18 with SCC) were classified as early-stage NSCLC patients, while 33 (11 with ADC, 15 with SCC and 7 with LCC) were classified as advanced-stage NSCLC patients. The Ethics Committee of the Medical University of Bialystok approved the study. Before collecting the samples, written informed consent for specimen collection was obtained from all participants.

All studied groups were age-, BMI- and sex-matched. The clinicopathological characteristics of the entire patients’ cohort is summarized in Table 1.

### 2.2. Tissue and Plasma Samples Preparation

Preparation of tissue samples was performed using the previously described method [22]. Lung tissue samples were homogenized in a freeze cold 50% methanol. Freezing cold acetonitrile was then added to extract the metabolites. After extraction, the samples were centrifuged, filtered and then analyzed.

Plasma samples preparation was performed using the previously described method [23]. The extraction of metabolites and simultaneous protein precipitation was carried out with a mixture of freezing cold methanol/ethanol (1:1). The samples were then centrifuged, filtered and analyzed.

The details of the samples preparation procedure are described in the Supplementary Materials.

### 2.3. Lung Tissue and Plasma Metabolic Fingerprinting

Metabolic fingerprinting was performed using the previously described LC-MS methods [22,23]. Samples were analyzed in four sets, plasma and tissue additionally divided into early and advanced NSCLC stages. Each tissue sample set was analyzed using reversed phase (RP) and hydrophilic interactions (HILIC) chromatography, while plasma samples using RP chromatography. Extracted samples were analyzed by an LC-MS system consisted of 1290 Infinity UHPLC (Agilent, Santa Clara, CA, USA) combined with 6550 iFunnel technology QTOF mass spectrometer as a detector (Agilent, Santa Clara, CA, USA). Analyses were performed in ESI+ and ESI− ion modes. Consequently, 12 data sets were obtained: early-stage tissue (RP+, RP−, HILIC+, HILIC−), advanced-stage tissue (RP+, RP−, HILIC+, HILIC−), early-stage plasma (RP+, RP−), and advanced-stage plasma (RP+, RP−). Data was collected in centroid mode at a scan rate of 1.5 spectra per second. Accurate mass measurements were obtained using calibrant solution delivery using a dual-nebulizer ESI source. Detailed information on performed LC-MS measurements is described in the Supplementary Materials.

### 2.4. LC-MS Data Treatment and Statistical Analysis

Raw data analysis was performed using Mass Hunter Qualitative Analysis Software B.06.00 (Agilent, Santa Clara, CA, USA). Alignment, data filtering and quality assurance protocol were performed for each of 12 data sets independently using Mass Profiler Professional 12.6.1 (Agilent, Santa Clara, CA, USA) software. Quality control (QC) samples were filtered to select features present in >50% of QCs and with relative standard deviation (RSD) for signal intensity in QCs <20%. Principal components analysis (PCA) was used to provide an overview of QCs projection in each data set. The tissue dataset was further normalized to an internal standard and the amount of protein in the sample. The details of raw data treatment are described in the Supplementary Materials. Two-groups comparisons (Control vs. ADC, SCC, or LCC as well as ADC vs. SCC) were performed using univariate statistics. Depending on the normality of the data distribution (assessed by the Shapiro–Wilk test), the t-test or non-parametric Mann–Whitney U-test was used. Obtained *p*-values were corrected by Benjamini–Hochberg false discovery rate (FDR). The level of statistical significance was set at 95% (*p* < 0.05). Univariate statistical analyses were performed in MATLAB (R2015a). Multivariate statistics were used to select compounds responsible for NSCLC subtypes separation. To select metabolites contributing the most into groups’ discrimination, partial least squares discriminant analysis (PLS-DA) was applied to log-transformed data. Statistically significant metabolites were selected based on the variable importance into projection (VIP) values (VIP > 1) and jackknife confidence interval (*p* < 0.05). Validation of the PLS-DA models was performed by cross-validation using the leave 1/3 out approach as described previously [24] or using a permutation test available in SIMCA−P + 13.0.3.0 (Umetrics, Umea, Sweden) software, which also was used for all multivariate data analysis.

### 2.5. Metabolite Identification

Accurate masses of significant features were searched against the METLIN, KEGG, LIPIDMAPS, and HMDB databases, which were simultaneously accessed by CEU Mass Mediator (http://ceumass.eps.uspceu.es/mediator/faces/index.xhtml, accessed on 21 September 2020) [25]. The identity of metabolites was confirmed by matching the experimental MS/MS spectra to MS/MS spectra from databases or fragmentation spectra and retention time obtained for the metabolite’s standard. Experiments were repeated with identical chromatographic conditions to the primary analysis. Ions were targeted for collision-induced dissociation (CID) fragmentation on the fly based on the previously determined accurate mass and retention time. Phospholipids and acylcarnitines were identified based on a previously described characteristic fragmentation pattern [26].

## 3. Results

### 3.1. Quality Assurance of Metabolomics Data

For each data set, a quality assurance protocol was performed. Close clustering of QC samples observed on PCA plots provided in the Supplementary Materials (Figures S1 and S2 obtained for tissue samples data and Figure S3 obtained for plasma samples data) indicate the proper quality of obtained data.

### 3.2. Samples Classification

**Figure 1:**
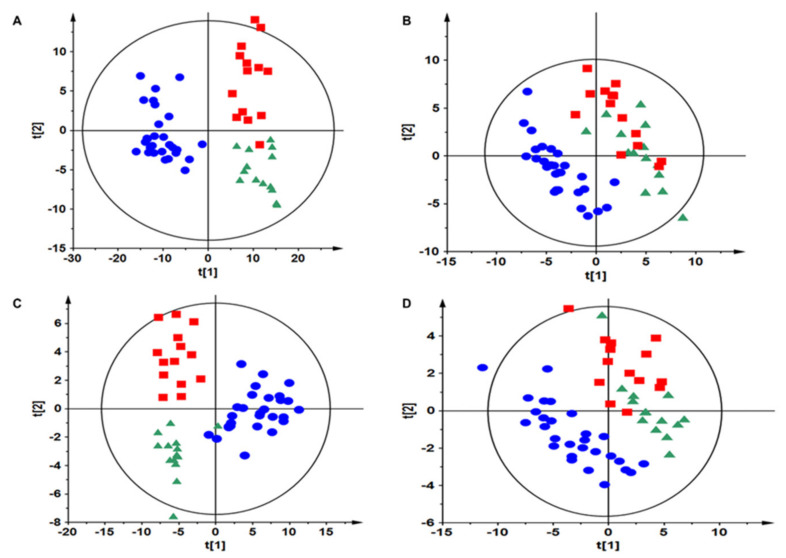
Discrimination between tumor and control tissue (early stage) as well as NSCLC subtypes based on lung tissue metabolic fingerprinting data. Data from RP and HILIC (ESI+ and ESI) methods were used to create these plots. PLS-DA plot showing discrimination between different NSCLC subtypes (ADC, SCC and control tissue (RP+)) is presented on panel (**A**) (Pareto scaling; cumulative values for six components: R^2^ = 0.983, Q^2^ = 0.853; *p*-value = 9.6 × 10^(−18)^). PLS-DA plot showing discrimination between different NSCLC subtypes (ADC, SCC and control tissue (RP−)) is presented on panel (**B**) (Pareto scaling; cumulative values for five components: R^2^ = 0.93, Q^2^ = 0.666; *p*-value = 2.2 × 10^(−15)^). PLS-DA plot showing discrimination between different NSCLC subtypes (ADC, SCC and control tissue (HILIC+)) is presented on panel (**C**) (Pareto scaling; cumulative values for three components: R^2^ = 0.858, Q^2^ = 0.732; *p*-value = 1.7 × 10^(−21)^). PLS-DA plot showing discrimination between different NSCLC subtypes (ADC, SCC and control tissue (HILIC−)) is presented on panel (**D**) (Pareto scaling; cumulative values for three components: R^2^ = 0.72, Q^2^ = 0.547; *p*-value = 4.1 × 10^(−7)^). ADC (green triangle), SCC (red square) and control (blue dots).

To classify the samples into studied groups, PLS-DA models were built. In the case of tissue samples collected from early-stage NSCLC patients, separation of the groups is presented in Figure 1. Advanced-stage tissue samples could be separated based on data obtained using RP chromatography (Figure S4) but not HILIC chromatography (data not shown). In the case of data collected for plasma samples, good models discriminating NSCLC subgroups were obtained for advanced-stage (Figure S5) but not for early-stage (data not shown) patients. Validation of the models indicated their proper quality to be used for multivariate statistics (Figure S6). The cross-validation results using the “leave 1/3 out” approach showed that excluded samples were classified correctly in 65 ± 13%.

### 3.3. Metabolomics of Tissue Samples

**Figure 2:**
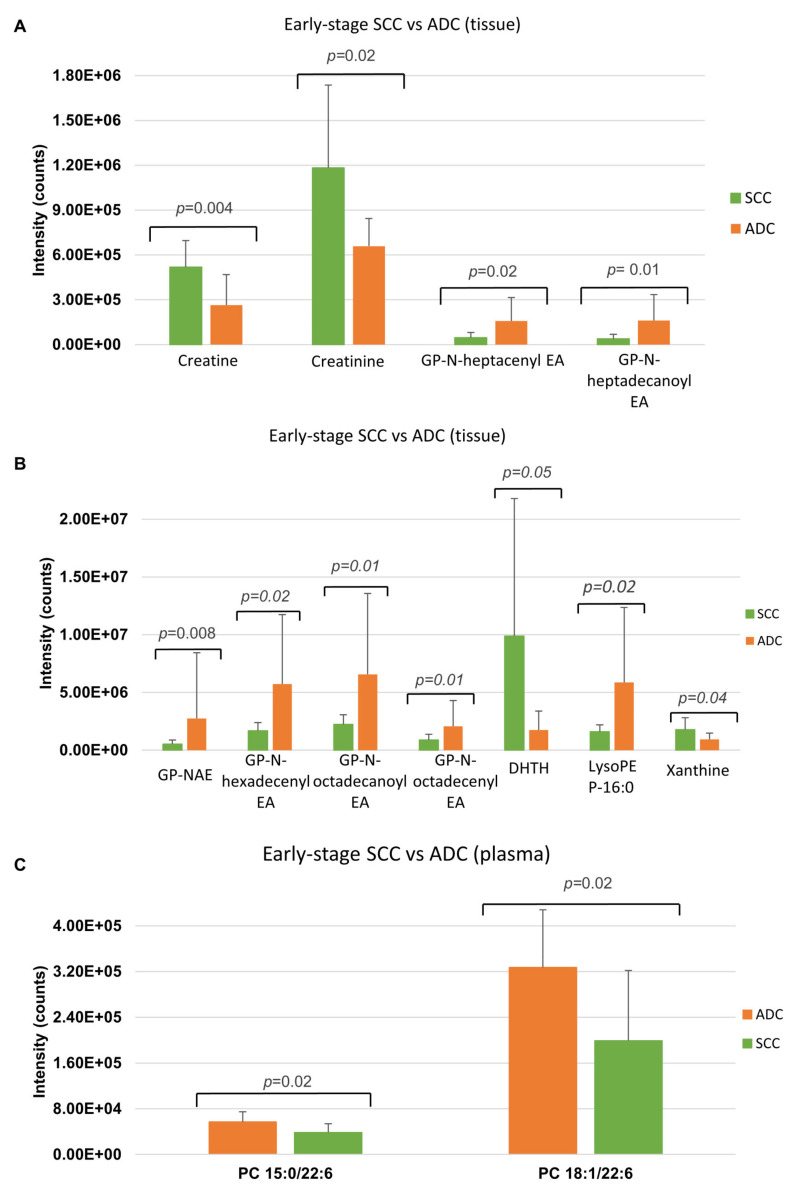
Metabolites that differentiate the early stages of SCC from ADC in tissue and plasma samples. Panels (**A**,**B**) show the intensity of selected identified tissue metabolites that differentiate the early SCC and ADC subtypes. Panel (**C**) shows the intensity of selected identified plasma metabolites that differentiate the SCC and ADCS subtypes (* *p*-value < 0.05; ** *p*-value < 0.01). Glycerophospho-N-heptacenyl ethanolamine (GP-N-heptacenyl EA); glycerophospho-N-heptadecanoyl ethanolamine (GP-N-heptadecanoyl EA); glycerophospho-(N-acyl)-ethanolamine (GP-NAE); glycerophospho-N-hexadecenyl ethanolamine (GP-N-hexadecenyl EA); glycerophospho-N-octadecanoyl ethanolamine (GP-N-octadecanoyl EA); and glycerophospho-N-octadecenyl ethanolamine (GP-N-octadecenyl EA), dihydrothymine (DHTH).

**Figure 3:**
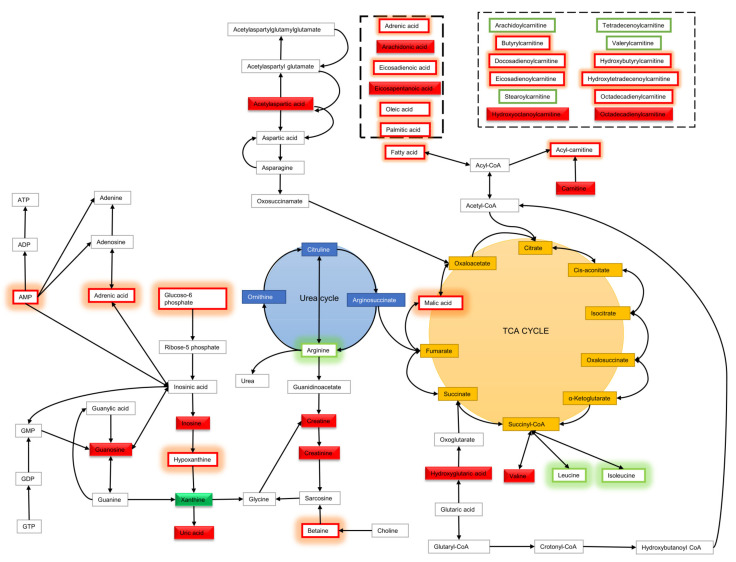
Summary of metabolic pathways which include metabolites discriminating early stages SCC and ADC tissue. Red rectangle—significant metabolites with a decreased percent of change in SCC vs. ADC comparison. Green rectangle—significant metabolites with an increased percent of change in SCC vs. ADC comparison. Red box—non-significant metabolites with a decreased percentage of change in the SCC vs. ADC comparison. Green box–non-significant metabolites with an increased percentage of change in SCC vs. ADC comparison.

**Figure 4:**
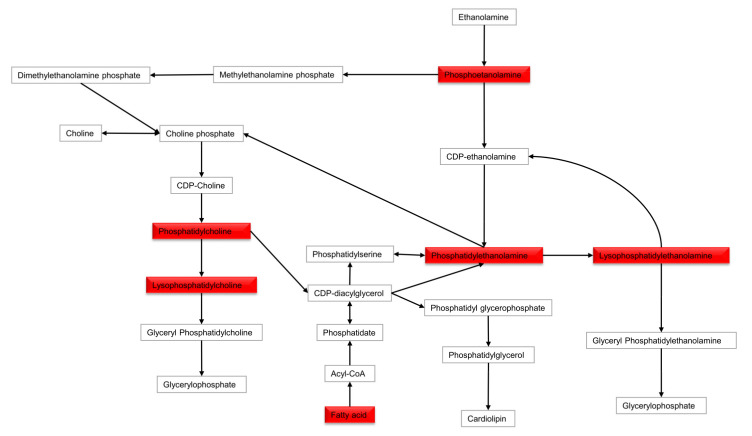
Pathway of glycerophospholipid metabolism. The metabolites in the red rectangle have a decreased percent of change in SCC vs. ADC comparison.

**Figure 5:**
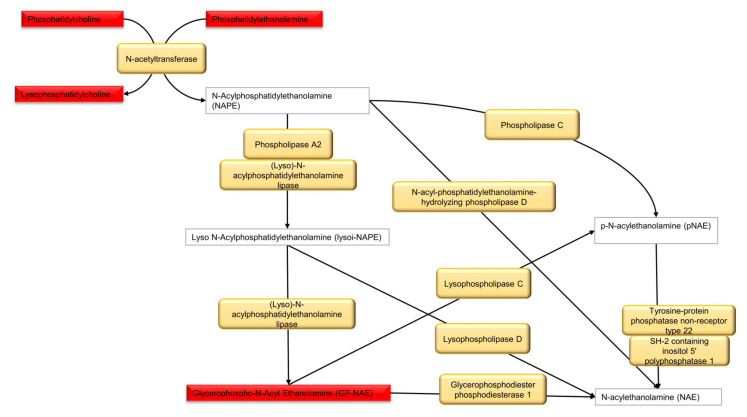
Pathway of endocannabinoid biosynthesis.

**Figure 6:**
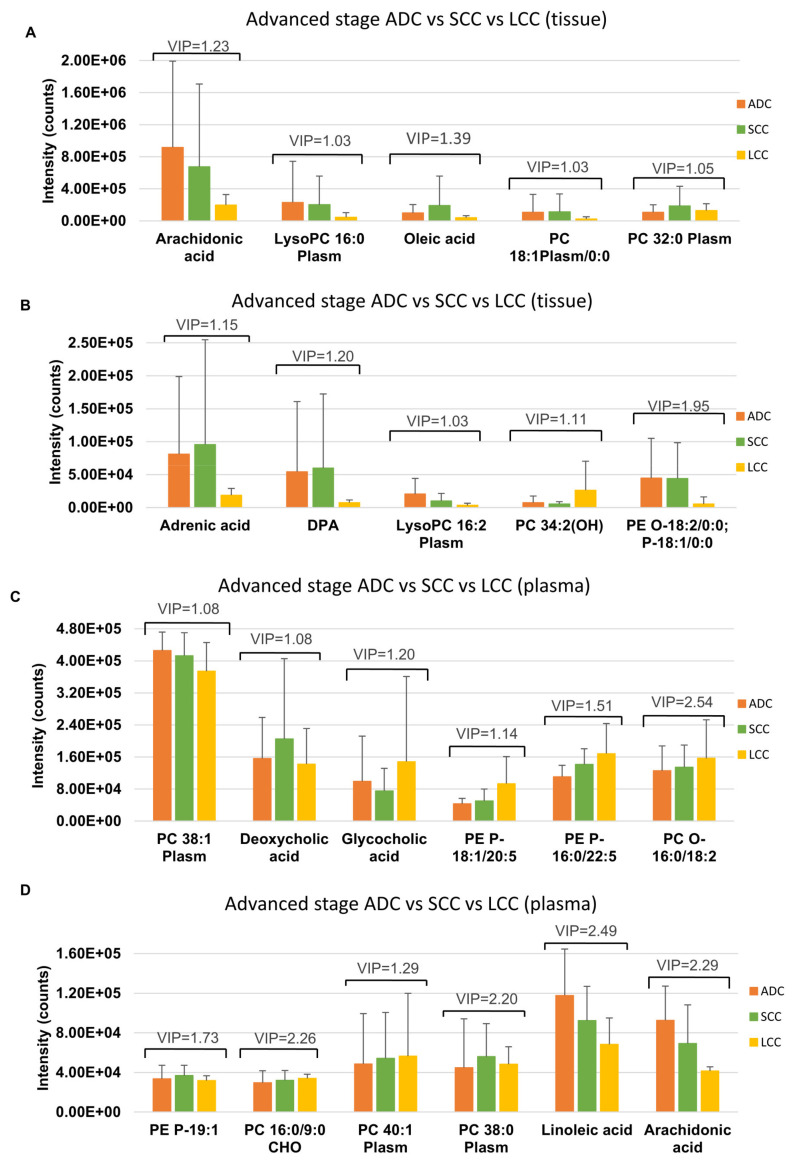
Metabolites that differentiate the advanced stages of SCC, ADC and LCC in tissue and plasma samples. Panels (**A**) and (**B**) show the intensity of selected identified tissue metabolites that differentiate the three subtypes in the advanced stage. Panels (**C**,**D**) show the intensity of selected identified plasma metabolites that differentiate the three subtypes in the advanced stage.

Metabolites discriminating tissue samples collected from NSCLC patients are presented in Supplementary Materials: Table S1 (early stage) and Table S2 (advanced stage). Metabolites significant at an early stage mainly belong to amino acids, fatty acids, carnitines, glycerophospholipids (GPL), sphingomyelins, plasmalogens GPL and others. Comparison of an early-stage SCC and ADC showed that creatine, creatinine, xanthine and dihydrothymine are upregulated in SCC, while metabolites belonging to fatty acids, carnitines, glycerophospholipids, lysoglycerophospholipids, amines, amino acids or amides are upregulated in ADC (Figure 2A,B). A summary of metabolic pathways which include metabolites discriminating early-stage SCC and ADC tissue is presented in Figure 3. Several of metabolites discriminating early-stage tissue samples belong to lipids. Among them, changes in a large number of glycerophospholipids were observed. A pathway of glycerophospholipid metabolism, on which metabolites significant in ADC vs. SCC comparison are highlighted, is presented in Figure 4. In addition, several glycerophospho-(N-acyl)-ethanolamines (GP-NAE) differentiating NSCLC subtypes have been identified. These metabolites, reported for the first time as discriminators of early-stage ADC and SCC tissues, are intermediates in endocannabinoid biosynthesis pathway (Figure 5). Comparing the results obtained for advanced-stage lung tissue samples (Table S2) with early-stage results, we see similar relationships in the groups of metabolites characterizing subtypes as in the early-stage samples. Carnitines, amino acids, lipids and fatty acids are the most important discriminators (Figure 6A).

The figure shows the biosynthesis of GP-NAE, metabolites discriminating early-stage SCC and ADC tissue samples. The metabolites in the red rectangle have a decreased percent of change in SCC vs. ADC and are statistically significant. The yellow rectangle present enzymes involved in the synthesis of endocannabinoids.

### 3.4. Metabolomics of Plasma Samples

Metabolites discriminating plasma samples collected from NSCLC patients are presented in Supplementary Materials: Table S3 (early-stage) and Table S4 (advanced stage). Analysis of plasma samples from patients with early NSCLC stage showed a much lower number of significant metabolites as compared to tissue results. The metabolites discriminating NSCLC from control group belong to GPL and sphingomyelins. Only two metabolites (PC 15:0/22:6 and 18:1/22:6) (Figure 2C) were found to be significant in SCC vs. ADC comparison at an early-stage. In the case of advanced-stage plasma samples, metabolites differentiating NSCLC subtypes mainly belong to fatty acids, carnitines and fatty acid amides (Figure 6A,B).

## 4. Discussion

Over the past decade, metabolomics was used in different studies to identify tissue, plasma or serum metabolites differentiating NSCLC subtypes. Regardless of the stage and subtype of non-small-cell lung cancer, most of these studies, especially those conducted on tissue, were performed on a relatively low number of cancer patients (*n* < 40) [14,17,20]. So far in only one study, both tissue and plasma from the same population were studied together. Berker et al. examined paired tissue and serum samples from 93 patients showing a panel of metabolites indicating the survival time of patients in the early stage of NSCLC [27].

This study aimed to identify metabolites in tissue and plasma samples that would allow the correct classification of NSCLC subtypes at an early and advanced stage. Moreover, the study aimed to compare the tissue and plasma metabolome of the same population, which may indicate systemic metabolic changes occurring in the body at various stages of NSCLC. Changes in the metabolites’ levels in both tissue and plasma of NSCLC patients and plasma of control group (patients with COPD) were investigated using the LC-MS technique.

Our research shows significant changes in many metabolites between control and cancerous tissue (both subtypes) at an early stage of the disease. Several metabolites have also been found different in comparison of SCC and ADC tissues (Table S1). Our results are in accordance with these published by Roch et al. [28] and Moreno et al. [29], who reported increased creatine/creatinine levels in SCC tissue compared to ADC. Increased creatine/creatinine levels in tumor tissue lead to increased ATP production, which is associated with the high-energy process of tumor growth and proliferation, especially in the early stages [11]. Elevated xanthine levels in stage I SCC have also been reported in other publications [29]. However, so far, the decrease in uric acid level in SCC tissue in comparison to ADC has not been reported. Such observation may suggest low xanthine oxidoreductase activity and reduced activity of the uric cycle in SCC, due to reduced levels of other purine bases such as inosine and guanosine. Similarly to Moreno et al. [29], in our study, we observed an increase in the level of dihydrothymine in SCC. In contrast, taurine levels were upregulated in ADC. Changes in systemic taurine levels can be used to predict the formation and malignant transformation of certain cancers. In addition, taurine has been shown to induce apoptosis and inhibit proliferation in breast cancer cells [30]. Recent studies on lung cancer cell lines have shown a significant reduction in the volume and weight of xenograft tumors in nude mice after taurine application [31]. Trimethylamine (TMA), increased in ADC in comparison to controls and significantly different between ADC and SCC in our study, is also a biologically interesting compound. TMA can be formed from L-carnitine (decreased in SCC vs. control and decreased in SCC vs. ADC in this study) or choline with support of different strains of bacteria [32]. Consequently, an increased level of TMA in ADC tissue may indicate an association of NSCLC, especially ADC subtype, with gut microbiota [33].

To date, a large number of scientific reports showing the great importance of fatty acids in the carcinogenesis process have been presented [34]. It is suggested that fatty acids belonging to the group of OMEGA-3 acids, such as docosahexaenoic acid (DHA), eicosapentaenoic acid (EPA) or eicosatrienoic acid (ETrE), show chemopreventive and therapeutic potential against lung cancer, inducing apoptosis of cancer cells especially in combination with platinum-based chemotherapy [35]. Comparison of cancer and control tissue clearly shows that GPL: lysophosphatidylcholines (LysoPC), lysophosphatidylethanolamines (LysoPE), lysophosphatidylinositols (LysoPI), PC and phosphatidylethanolamines (PE) as well as acylcarnitines are accumulated in cancer tissue, but this effect is stronger for ADC subtype, with some GPL discriminating ADC and SCC tissue (Table S1). Changes in the lipids, which are used as an energy source (acylcarnitines) and building components (phospholipids) for rapidly multiplying cancer cells, are very often observed in both tissue and plasma studies on NSCLC [36,37,38,39]. Changes in plasmalogens e.g., (LysoPC P-16:0; LysoPE P-16:0 and P-17:0; PE P-18:0/20:4; PC P-16:0/18:2, PC P-16:0/20:5 and PC O-16:1/18:2) are especially interesting (Tables S1 and S3). So far, the role of plasmalogens has been described based on tissue samples analysis [40], while the role in plasma of NSCLC patients has not yet been described. To date, the occurrence of plasmalogens in cancer has been quite accurately presented, among others, in gastrointestinal [41] and breast cancer [42] in various biological materials. Plasmalogens play a crucial role as endogenous antioxidants, protecting phospholipid, lipid and lipoprotein molecules against oxidative stress. The plasmalogens decomposition protects polyunsaturated fatty acids and other membrane lipids from oxidation. Thus, plasmalogens rather interfere with the multiplication stage than the initiation of lipid peroxidation, which has a considerable role in inhibiting the peroxidation of polyunsaturated fatty acids and low-density lipoproteins oxidation [41]. We observed a statistically significant decrease in the level of plasmalogens in plasma of patients with early NSCLC in comparison to control group, while in cancer tissue, we found an increase in these metabolites in comparison to normal tissue. Accumulation of plasmalogens in the early-stage cancer tissue may play a protective role against the oxidative activity during cancer development. Considering metabolites discriminating ADC and SCC tissue, in our study for the first time, differences in several GP-NAE are reported (Table S1). GP-NAE are constituents of N-Acyl ethanolamines (NAEs) biosynthesis [43]. NAEs are an important class of signaling lipids involved, among others, in inflammation and the regulation of TNFα [44]. Among NAEs, so far, the role of anandamide and 2-arachidonoylglycerol in cancer has been thoroughly studied [45]. The reduction of NAE in SCC vs. ADC requires further research that may clarify the metabolic mechanisms that differentiate these two subtypes at an early stage. Among other metabolites discriminating early-stage ADC and SCC tissues, acetylaspartic acid can be mentioned. Lou et al. detected N-acetyl aspartate (NAA) in NSCLC, while in the normal pulmonary epithelium, it was not detected. Increased N-acetylaspartate synthetase (Nat8l) expression was also confirmed in approximately 40% of examined ADC and SCC cases. It has been suggested that NAA biosynthesis depends on the availability of glutamine, which is the main carbon source for the NAA molecule in NSCLC cells [46]. In addition, Fahrmann et al. presented the results of increasing the glutaminolysis process, especially in ADC [19].

Only few plasma metabolites, mainly PC and LysoPC, were found to be significant in comparison of the control group and early-stage NSCLC patients (mainly in comparison of the control group with SCC group). However, contrary to other metabolomics studies on the plasma of NSCLC patients [38,47], in our study, the control group was composed of patients with COPD what is crucial to exclude metabolites related to inflammation which may falsely indicate cancer [48]. Even though, metabolites from the same classes were proposed as early-stage NSCLC plasma markers by other authors [10,38,47], but in these studies, a larger number of discriminating metabolites and with a higher magnitude of change was observed. As these metabolites are known to be affected by inflammatory processes [49], observed differences between other studies and ours can be explained by the different control group selected.

Several of the significant metabolites observed in our study have already been introduced as potential NSCLC classifiers. Diacetylspermine has been identified in plasma as a useful biomarker for early detection of NSCLC while in urine as a prognostic marker [50,51]. Carnitines or lipids have also been noted as discriminators of cancerous tissue from control one [10,29,36].

Although the number of significant metabolites discriminating advanced-stage NSCLC subtypes is much lower than in early stage, similarly, carnitines, amino acids, lipids and fatty acids are the most important discriminators. Amino acids, such as histidine and arginine, have the highest intensity in the SCC subtype. Ni et al. presented the use of arginine, among others, as a biomarker of lung cancer [12]. Changes in amino acids indicate increased L-type amino acid transporter 1 activity, which has also been noted in other studies [52]. We noted the same with acylcarnitines, which are the most intense in SCC tissue. This observation may indicate very intense processes occurring in mitochondria (acetyl-CoA synthesis) to obtain the energy for a highly proliferating tumor. The accumulation of acylcarnitines in SCC tumor is also reflected by their level in plasma. The intensity of acylcarnitines is lower in SCC patients’ plasma compared to ADC patients and similar to those with LCC subtype (Table S4). We also observed significant differences between the NSCLC subtypes in the level of such long-chain fatty acids such as adrenic or oleic acid (Figure 6). Their highest intensity in SCC tissue may indicate greater use of the long-chain acyl-CoA synthetase, primarily ACSL3 and ACSL4 in advanced SCC [34]. Only arachidonic acid was less intense in SCC and LCC advanced tissue. Similarly to previously described acylcarnitines, we observe reduced levels of long-chain fatty acids (linoleic and arachidonic) in plasma of advanced SCC patients compared to ADC, but not LCC patients (Table S4). In plasma of advanced-stage SCC patients, the intensity of one bile acid (deoxycholic) was found to be the highest in comparison to other subtypes. Bile acids are responsible for cholesterol homeostasis and are central signaling molecules transmitting messages on the consumption and availability of energy to peripheral tissues. This observation, together with previously mentioned results on fatty acids and acylcarnitines, may indicate a higher energy demand in SCC subtype in comparison to ADC and LCC. Furthermore, it also suggests stronger expression of TGR5 receptor in SCC tissue, which promotes cell migration and invasion through a TGR5-dependent way [53].

In the case of lipids, we have observed reduced levels of LysoPC, especially LysoPC 18:3 in plasma of cancer patients compared to the control group in the early stage. By contrast, tissue levels of all LysoPC were clearly increased in all types of cancer tissue compared to control tissue. The reduced level of all LysoPC at an early NSCLC stage was also observed by other researchers [38]. Low levels of LysoPC can be associated with a higher rate of LysoPC and LysoPC-related fatty acids consumption by cancer cells [54] as well as by an increased extracellular LysoPC cleavage rate [55]. In addition, scientific reports suggest that LysoPC participate in the process of phagocyte recruitment and the opsonization of apoptotic cells [54]. The same situation is also observed for LysoPE but only for patients with ADC. By contrast, the level of LysoPE in patients with SCC does not change in plasma, relative to the tissue. Additionally, what is very interesting, in patients with advanced LCC, the intensity of LysoPE in plasma increases, while in the tissue, it decreases, unlike in patients with ADC.

The use of LysoPE as potential biomarkers in the early stages of NSCLC has already been demonstrated [47]. Moreover, LysoPE levels may be associated with lysophosphatidylethanolamine acyltransferase 2 (LPEAT2) activity. Studies conducted on neuronal cells indicate an important role of LPEAT2 in the incorporation of DHA into phospholipids in the brain via the remodeling pathway. Furthermore, overexpression of the enzyme (LPEAT2) caused cell death when neural cells were treated with DHA [56]. In addition, the previously described role of DHA as a modulator of cell death in combination with chemotherapy [35,57], which we identified at an early-stage, can confirm the presented theory. In the same way in patients at an early stage and at an advanced stage, we observed an increase in the number of identified PC and PE. In patients with LCC, most of the plasma PC is more intense than in patients with ADC and SCC. By contrast, the intensity of plasmalogens in tissue increases in patients with ADC and SCC compared to LCC. We also identified oxidized lipids in both tissue and plasma, with the intensity of the oxidized lipids decreasing in plasma and tissue of patients with ADC and SCC (Tables S2 and S4). A significant increase in sphingomyelin and sphingosine-1-phosphate (S1P) intensity was observed in ADC patients’ plasma. This confirms the increase in sphingomyelin synthase (SMS) enzymes, which convert ceramide to sphingomyelin (SM) via insertion of a choline moiety into ceramide as a head group using PC as a donor [58], which is also indicated by the significantly reduced intensity of some PC in the plasma of ADC patients. The increased distribution of ceramides, especially in patients with ADC, may also indicate an increased level of S1P. The role of ceramides and S1P in lung cancer is quite well described [59].

## 5. Conclusions

In summary, we observed a significantly changed metabolic profile in the early and advanced stage of NSCLC. However, in the case of early-stage patients, stronger discrimination was observed based on tissue metabolites, while in the case of advanced-stage patients, it was based on plasma metabolites. Using untargeted metabolomics, we showed for the first time that glycerophospho (N-acyl) ethanolamines discriminate early-stage ADC and SCC tissue. In addition, performed analyses enabled us to point out the differences between plasma of NSCLC and COPD patients and indicate that plasmalogens are potential early-NSCLC plasma biomarkers. Our research also showed new biochemical changes occurring in NSCLC and confirmed the results presented so far. Among others, significant metabolites indicate the possible role of gut microbiota in the formation and progress of NSCLC. Presented research emphasizes the power of metabolomics in identifying potential biomarkers and exploration of the mechanisms underlying NSCLC.

## Figures and Tables

**Table 1:** Patients’ characteristics.

Type of Biological Material	Patients Characteristic	Squemous Cell Carcinoma (SCC)	Adenocarcinoma (ADC)	Large Cell Carcinoma (LCC)
Tissue	Age (mean)	64.45 ± 8.02	64.77 ± 8.44	64.58 ± 6.43
	BMI (mean)	25.4 ± 3.48	25.4 ± 3.59	25.5 ± 2.83
	Male	39	23	10
	Female	15	10	2
	pTNM: I A	7	8	0
	I B	7	6	0
	II A	13	6	2
	II B	18	7	7
	III A	9	6	3
Plasma	Age (mean)	65.61 ± 6.52	64.16 ± 6.91	64.57 ± 5.62
	BMI (mean)	27.4 ± 4.48	26.0 ± 3.41	24.9 ± 1.78
	Male	21	20	6
	Female	12	12	1
	pTNM: I A	10	10	0
	I B	9	10	0
	II A	3	4	0
	II B	7	6	6
	III A	4	2	1
	Control group			
Plasma	Number of patients	20		
	Age (mean)	61.5 ± 12.06		
	Male	13		
	Female	7		

## Data Availability

The datasets analyzed during the current study are available from the corresponding author on reasonable request.

## Supplementary Materials

The following are available online at https://www.mdpi.com/article/10.3390/cancers13133314/s1, Figure S1: Clustering of QC samples in early- and advanced-stage tissue samples, Figure S2: Clustering of QC samples in early- and advanced-stage tissue samples, Figure S3: Clustering of QC samples in early- and advanced-stage plasma samples, Figure S4: Discrimination between NSCLC subtypes based on lung tissue (advanced-stage) metabolic fingerprinting data; Figure S5: Discrimination between NSCLC subtypes based on plasma samples (advanced stage) metabolic fingerprinting data, Figure S6: Permutation test showing the reliability of the separation obtained in the multidimensional model (PLS-DA) in advanced NSCLC stage, Table S1: Tisuse early stage, Table S2: Tisuse advanced stage, Table S3: Plasma early stage, Table S4: Plasma advanced stage, Supplementary Materials.

## Abbreviations

ADC	Adenocarcinoma
ALK	Tyrosine kinase receptor
BMI	Body Mass Index
CID	Collision-induced dissociation
cMYC	C-myc protein
COPD	Chronic obstructive pulmonary disease
DHA	Docosahexaenoic acid
EGFR	Epidermal growth factor receptor
EML4-ALK	Tyrosine-protein kinase receptor
EPA	Eicosapentaenoic acid
ESI−	Electrospray ionization negative
ESI+	Electrospray ionization positive
ETrE	Eicosatrienoic acid
FDR	False discovery rate
GPL	Glycerophospholipids
GP-NAE	Glycerophospho (N-acyl) ethanolamines
HILIC	Hydrophilic interactions chromatography
KRAS	GTPase KRas
LCC	Large cell cancer
LC-MS	Liquide chromatography mass spectrometry
LPEAT2	Lysophosphatidylethanolamine acyltransferase 2
LysoPC	Lysophosphatidylcholines
LysoPE	Lysophosphatidylethanolamines
LysoPI	Lysophosphatidylinositols
NAA	N-acetyl aspartate
NRF2	Nuclear factor erythroid 2-related factor 2
NSCLC	Non-small-cell lung cancer
PC	Phosphatidylcholines
PCA	Principal components analysis
PE	Phosphatidylethanolamines
PLS-DA	Partial least squares discriminant analysis
QC	Quality control
ROS1	Proto-oncogene tyrosine-protein kinase ROS
RP	Reversed-phase chromatography
SCC	Squamous cell cancer
SM	Sphingomyelin
TCA cycle	Tricarboxylic acid cycle
TMA	Trimethylamine
TNM	Tumor, node, metastasis classification
TP53	Tumor protein p53
VIP	Variable importance into projection values
