# Supplementary material for: The Ability of Metabolomics to Discriminate Non-Small-Cell Lung Cancer Subtypes Depends on the Stage of the Disease and the Type of Material Studied
Source: Cancers (Basel). 2021 Jul 1;13(13):3314. doi: 10.3390/cancers13133314 (PMC8268630; doi:10.3390/cancers13133314)
Supplement: Supplementary file 1 [file cancers-13-03314-s001.zip › cancers-1248054-Supplementary .pdf]

cancers

MDPI

Supplementary Files

The Ability of Metabolomics to Discriminate Non-Small-Cell Lung Cancer Subtypes Depends on the Stage of the Disease and the Type of Material Studied

Tomasz Kowalczyk, Joanna Kisluk, Karolina Pietrowska, Joanna Godzien, Miroslaw Kozlowski, Joanna Reszeć, Ewa Sierko, Wojciech Naumnik, Robert Mróz, Marcin Moniuszko, Adam Kretowski, Jacek Niklinski and Michal Ciborowski

1. Metabolic fingerprinting of plasma samples

1.1. Plasma Samples Preparation

On the day of analysis, the samples were thawed on ice. Protein precipitation and extraction of metabolites was performed by vortex mixing (for 1 minute) of one volume of plasma sample with three volumes of freeze cold (-20°C) methanol/ethanol (1:1). After extraction, the samples were stored on ice for 10 minutes and centrifuged at 21,000 x g for 20 minutes at 4°C. The supernatant was filtered through a 0.22 µm nylon filter into glass vials. Quality control (QC) samples were prepared by mixing equal volumes of all samples. The resulting mixture was prepared according to the same procedure as the other samples.

1.2. LC-MS analysis of Plasma Samples

Samples were analyzed using the 1290 Infinity UHPLC system combined with 6550 iFunnel technology QTOF mass spectrometer as a detector (both Agilent Technologies). Analyses were performed in ESI+ and ESI− ion modes. One microliter of a sample was injected into a thermostated (60°C) Zorbax Extend-C18 RRHT (2.1×50 mm, 1.8-µm particle size, Agilent Technologies) chromatographic column. The flow rate was 0.6 mL/min with solvent A (deionized water with 0.1% formic acid) and solvent B (acetonitrile with 0.1% formic acid). The chromatographic gradient started at 5% of phase B for the first minute. Next, the mobile phase composition was changed by increasing phase B to 80% (from 1 to 7 minutes) and to 100% (from 7 to 11.5 minutes). Following that, the system was re-equilibrated by reverting phase composition to initial conditions (5% phase B) in 0.5 minutes, which was kept from 12 to 15 minutes. The mass spectrometer was operated in full scan mode. Data were acquired from 50 to 1000 *m/z* range separately in positive and negative ion modes at the scan rate of 1.5 scans per second. Accurate mass measurements were obtained using calibrant solution delivery using a dual-nebulizer ESI source. A calibrating solution (G1969-85000) containing reference masses at *m/z* 121.0509 (protonated purine) and *m/z* 922.0098 (HP-921) in the positive ion mode or *m/z* 119.0363 (proton abstracted purine) and *m/z* 966.0007 (formate adduct of HP-921) in the negative ion mode was continuously introduced with an isocratic pump (Agilent, Santa Clara, California, USA) at a flow rate of 0.5 mL/min (1:100 split). Nebulizer pressure was set at 52 psig, nozzle voltage at 1000 V, and capillary voltages were set at 3000 and 4000 V in the positive and negative ion mode, respectively.

2. Metabolic fingerprinting of tissue samples

2.1. Tissue sample preparation

Ten milligrams of lung tissue sample was placed in Eppendorf tube together with 2 stainless steel beads (5 mm) and 200 µL of freeze cold (-20°C) 50% methanol. Bead mill homogenizer (Tissue Lyser LT; Qiagen Hilden, Germany) was used for sample

homogenization by 8 min (30 Hz). After homogenization beads were removed, 200 µL of freeze cold (-20°C) acetonitrile containing 1ppm of zomepirac (internal standard) was added to the sample. Metabolites were extracted by vortex-mixing of the samples for 1h. After extraction, samples were centrifuged (Eppendorf, Hamburg, Germany) at 21,000 × g for 20 minutes at 20°C. After centrifugation the supernatant was filtered through a 0.22 µm nylon filter (ThermoFisher Scientific, Waltham, Massachusetts, USA) and equal volumes of each sample were pooled to get a quality control (QC) sample. The remaining volume was divided into two parts, one for LC-RP-MS analysis and second (diluted 1:1 with acetonitrile) for LC-HILIC-MS analysis. Blank extraction (prepared following the same procedure as biological samples but not containing tissue) was also prepared and analyzed together with biological samples.

2.2. LC-RP-MS Analysis of Tissue Samples

Extracted sample (1µL) was injected into a thermostated (60°C) reversed-phase Zorbax Eclipse Plus C8 RRHD 2.1x150 mm, 1.8 µm column (Agilent Technologies, Santa Clara, California, USA). The flow rate was 0.6 mL/min with solvent A (water with 0.1% formic acid) and solvent B (acetonitrile with 0.1% formic acid). The gradient started at 25% phase B and was increasing to reach 95% of phase B in 14 min. This proportion was kept for 1 min and after that, the gradient returned to starting conditions (25% of phase B) in 0.1 min and was maintained at this solvents proportion for 4.9 min in order to re-equilibrate the system for the next injection. The mass spectrometer was operated in full scan mode from *m/z* 50–1000. The capillary voltage was set to 3 kV for ESI+ and 4kV for ESI- mode; the drying gas flow rate was 12 L/min at 250°C and gas nebulizer at 52 psig; fragmentor voltage was 250 V for both ESI modes. Measured *m/z* values were corrected with the use of two reference masses at m/z 121.0509 (protonated purine) and *m/z* 922.0098 (HP-921) in positive ion mode or *m/z* 119.0363 (proton abstracted purine) and *m/z* 966.0007 (formate adduct of HP-921) in negative ion mode. Reference mass solution was continuously introduced by an isocratic pump (Agilent, Santa Clara, California, USA) at a flow rate increasing from 0.25 mL/min to 0.5 mL/min (1:100 split) from 0 to 15 min and back to 0.25 mL/min in 5 min in positive ion mode and from 0.5 mL/min to 0.75 mL/min (1:100 split) from 0 to 15 min and back to 0.5 mL/min in 5 min in negative ion mode.

2.3. LC-HILIC-MS analysis of tissue samples

Extracted sample was diluted twice with acetonitrile and 0.5 µL of diluted sample was injected into a thermostated (30°C) Poroshell Hilic 2.1x100 mm, 2.7 µm column (Agilent Technologies, Santa Clara, California, USA). The flow rate was 0.1 mL/min with solvent A (10mM ammonium formate in water, pH=4) and solvent B (acetonitrile with 0.1% formic acid). The gradient started at 70% phase B and was decreasing to reach 60% of phase B in 8 min. Subsequently, to clean the column, the gradient was increasing to 95% of phase B in 0.1 min and this proportion was kept for 0.8 min. After that the gradient returned to starting conditions (70% of phase B) in 0.1 min and was maintained at this solvents proportion for 3 min to re-equilibrate the system for the mass spectrometer was operated in full scan mode from *m/z* 50–350. The capillary voltage was set to 3.5 kV for both ESI modes; the drying gas flow rate was 13 L/min at 200°C and gas nebulizer at 30 psig; fragmentor voltage was 200 V for both ESI modes. Measured *m/z* values were corrected with the use of two reference masses at *m/z* 121.0509 (protonated purine) and *m/z* 322.0481 (HP-0321) in positive ion mode or *m/z* 112.9855 (TFA anion) and *m/z* 301.9981 (HP-0285) in negative ion mode, which were continuously introduced by an isocratic pump (Agilent, Santa Clara, California, USA) at a flow rate of 0.4 mL/min or 0.8 mL/min (1:100 split) in ESI+ or ESI- modes, respectively.

3. LC-MS Data Treatment

The raw data collected by the analytical instrumentation was cleaned of background noise and unrelated ions by the Molecular Feature Extraction (MFE) tool in Mass Hunter Qualitative Analysis Software B.06.00 (Agilent, Santa Clara, California, USA). The MFE creates a list of all possible compounds described by mass, retention time (RT), and abundance. The limit for the background noise was set to 2000 counts for data extraction by MFE and the following adduct settings were applied to identify co-eluting adducts of the same feature: +H, +Na, +K in positive ion mode and −H, +HCOO, +Cl for negative ion mode. Dehydration neutral losses were also allowed. Additionally +NH4 was included in the list of possible adducts for data recorded in HILIC ESI+ mode. Alignment and data filtering were performed using Mass Profiler Professional 12.6.1 (Agilent, Santa Clara, California, USA). Parameters applied for the alignment were 1% for RT and 15 ppm for the mass variation. Before statistical analysis data was filtered to keep only the metabolic features with CV < 20% in QC samples and present in at least 80% of the samples in at least one of the biological groups being compared.

Supplementary figures:

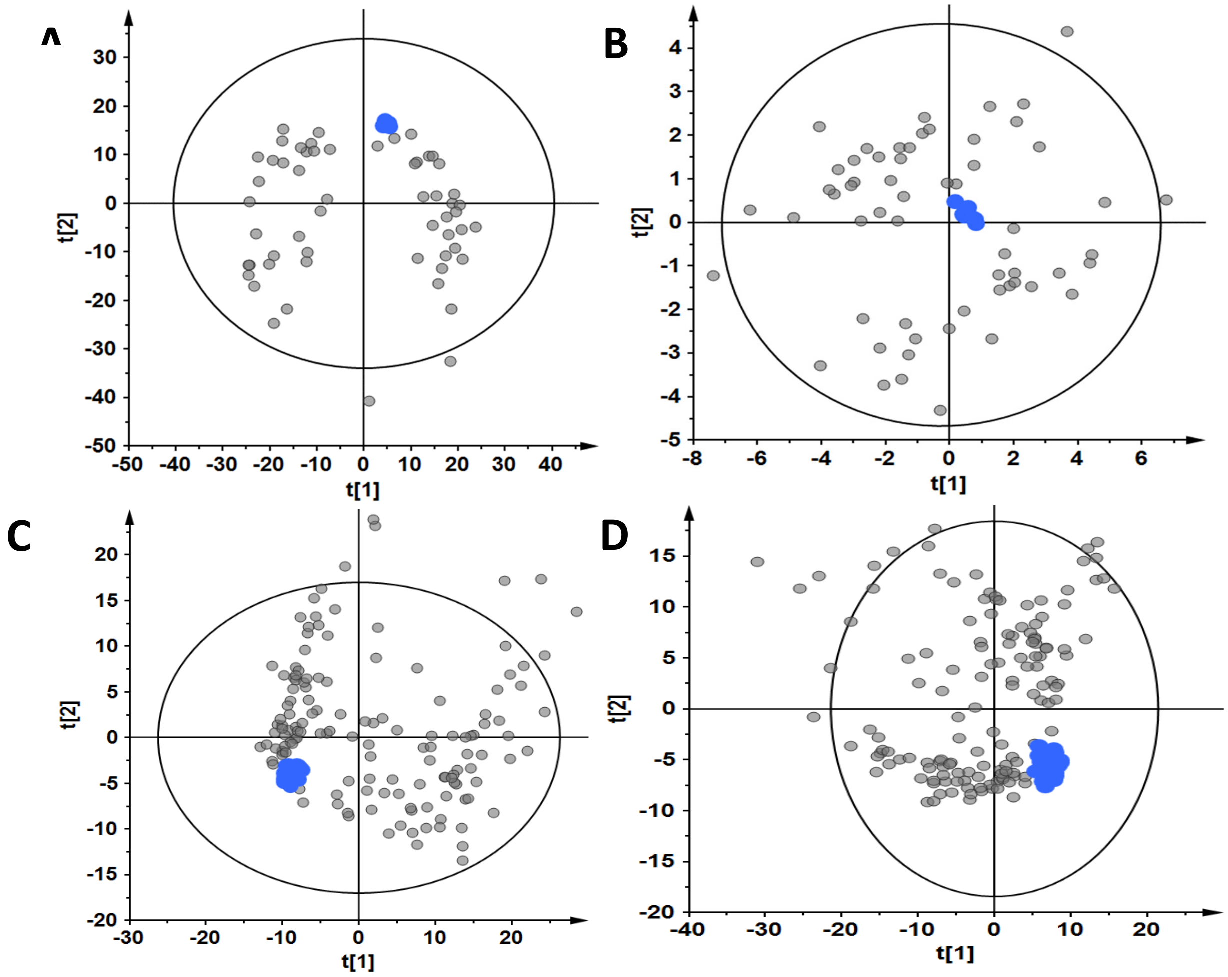

Figure S1. Clustering of QC samples in early- and advanced-stage tissue samples. PCA plots showing clustering of QC samples. Panel A: early-stage, RP chromatography, ESI+; Panel B: early-stage, RP chromatography, ESI-; Panel C: advanced-stage, RP chromatography, ESI+; Panel D: advanced-stage, RP chromatography, ESI-. QC samples (blue dots), biological samples (grey dots).

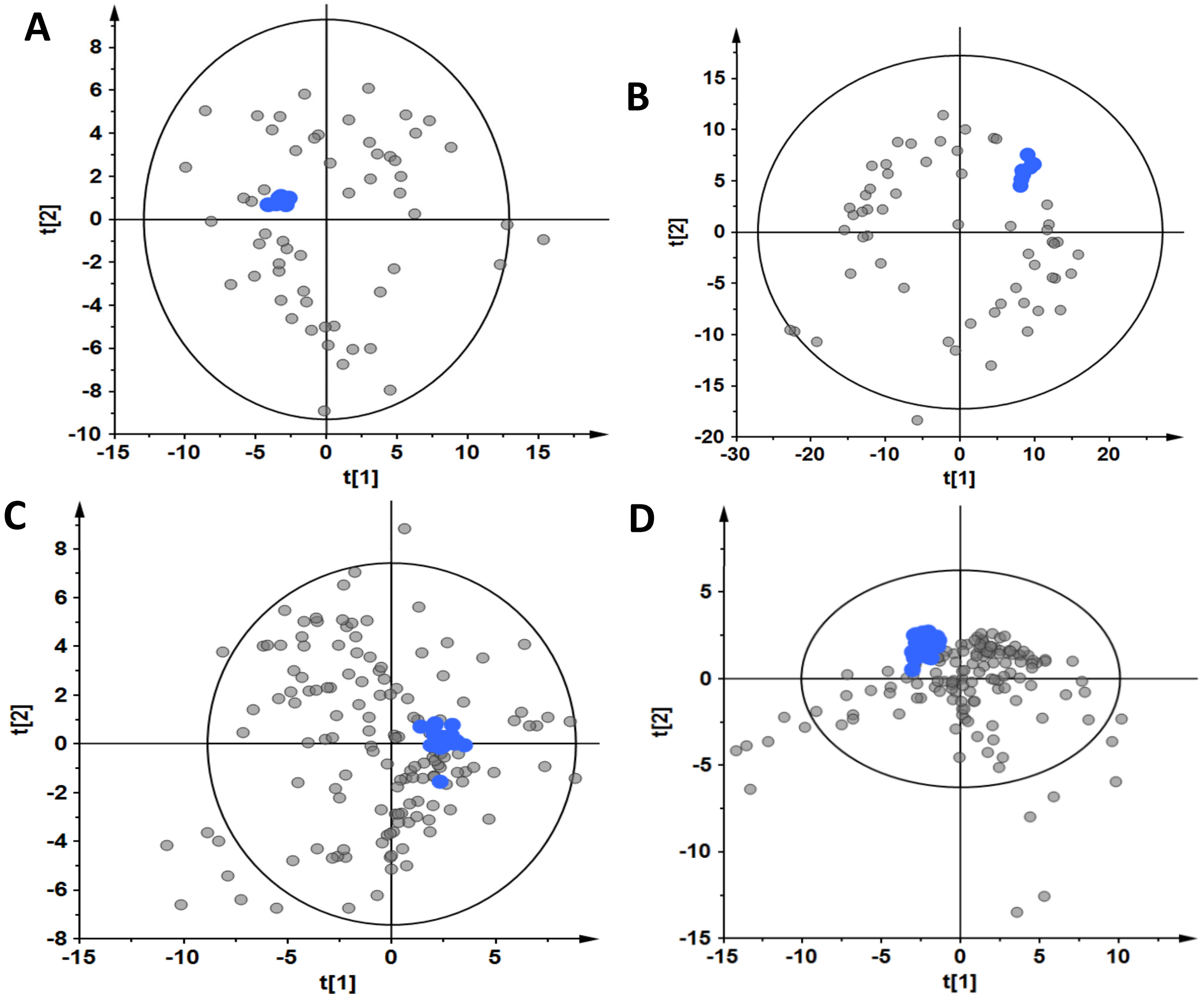

Figure S2. Clustering of QC samples in early- and advanced-stage tissue samples. PCA plots showing clustering of QC samples. Panel A: early-stage, HILIC chromatography, ESI+; Panel B: early-stage, HILIC chromatography, ESI-; Panel C: advanced-stage, HILIC chromatography, ESI+; Panel D: advanced-stage, HILIC chromatography, ESI-. QC samples (blue dost), biological samples (gray dots).

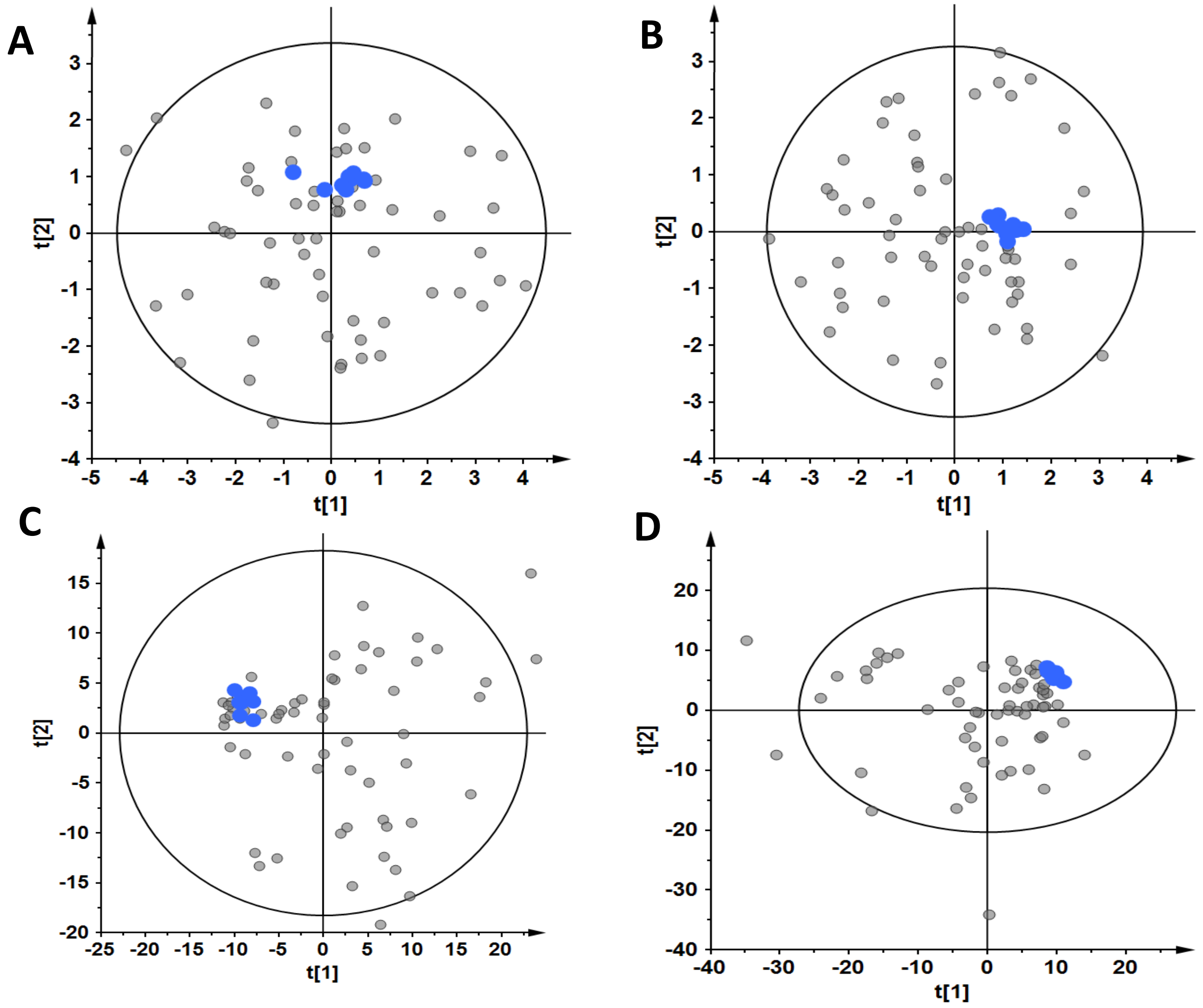

Figure S3. Clustering of QC samples in early- and advanced-stage plasma samples. PCA plots showing clustering of QC samples. Panel A: early-stage, RP chromatography, ESI+; Panel B: early-stage, RP chromatography, ESI-; Panel C: advanced-stage, RP chromatography, ESI+; Panel D: advanced-stage, RP chromatography, ESI-. QC samples (blue dost), biological samples (gray dots).

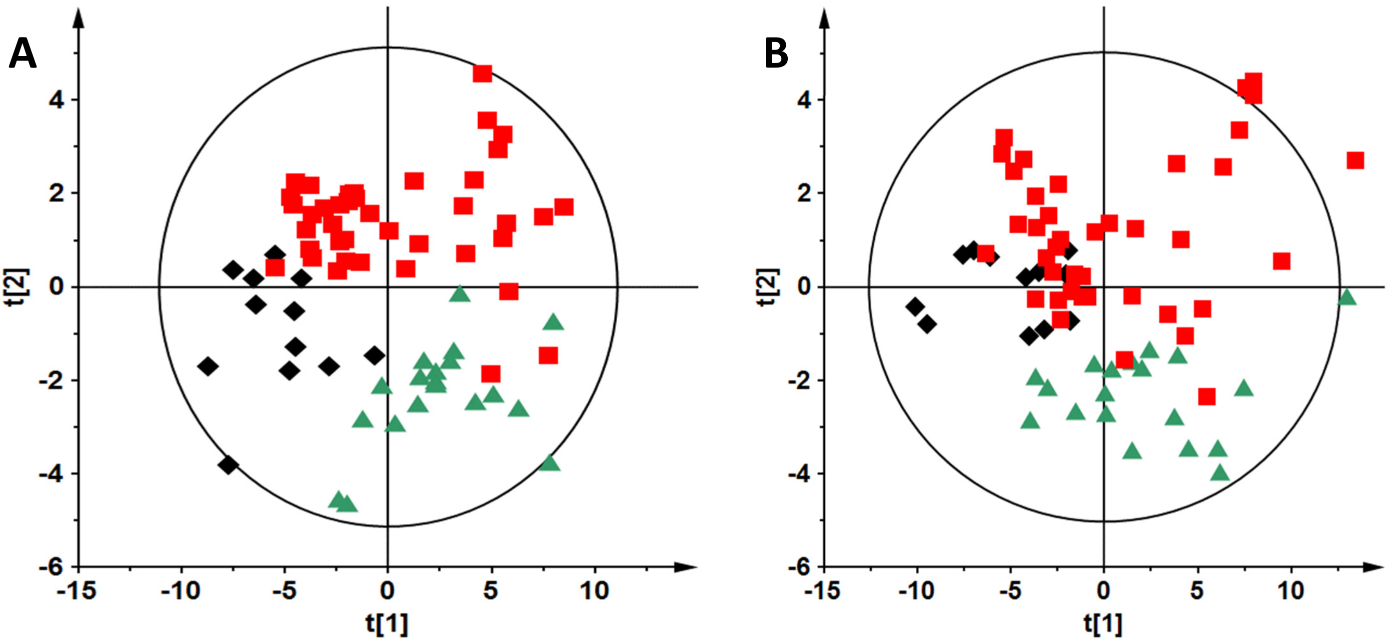

Figure S4. Discrimination between NSCLC subtypes based on lung tissue (advanced-stage) metabolic fingerprinting data. Data from RP (ESI+ and ESI-) method were used to create these plots. PLS-DA plot showing discrimination between different NSCLC subtypes: ADC, SCC, LCC (RP+) is presented on panel A (Pareto scaling, cumulative values for 6 components: R2=0.913, Q2=0.683; *p*-value=0.000002). PLS-DA plot showing discrimination between different NSCLC subtypes: ADC, SCC, LCC (RP-) is presented on panel B (Pareto scaling, cumulative values for 2 components: R2=0.408, Q2=0.256; *p*-value=5.4x10(-14)). ADC (green triangles), SCC (red squares), LCC (black diamonds).

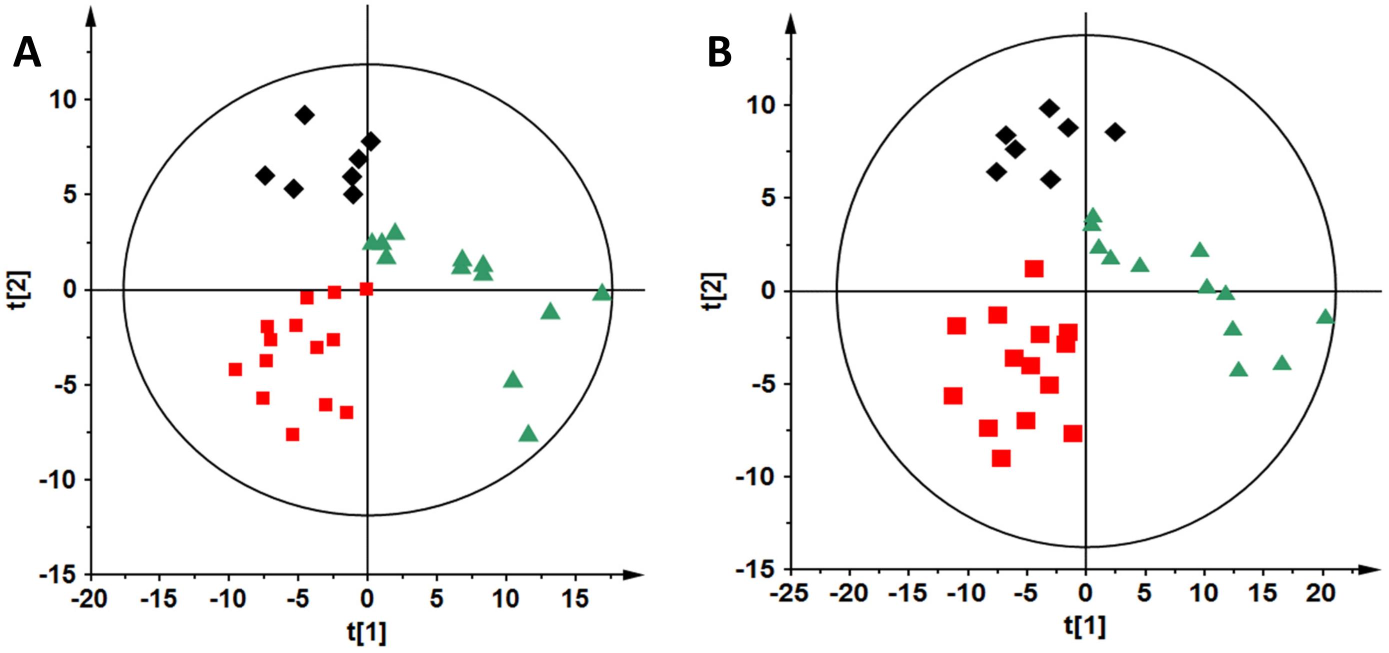

Figure S5. Discrimination between NSCLC subtypes based on plasma samples (advanced stage) metabolic fingerprinting data. Data from RP (ESI+ and ESI-) methods were used to create these plots. PLS-DA plot showing discrimination between different NSCLC subtypes: ADC, SCC, LCC (RP+) is presented on panel A (Pareto scaling, cumulative values for 2 components: R2=0.698, Q2=0.22; p=0.05). PLS-DA plot showing discrimination between different NSCLC subtypes: ADC, SCC, LCC (RP-) is presented on panel B (Pareto scaling, cumulative values for 5 components: R2=0.984, Q2=0.646; p=0.06). ADC (green triangles), SCC (red squares), LCC (black diamonds).

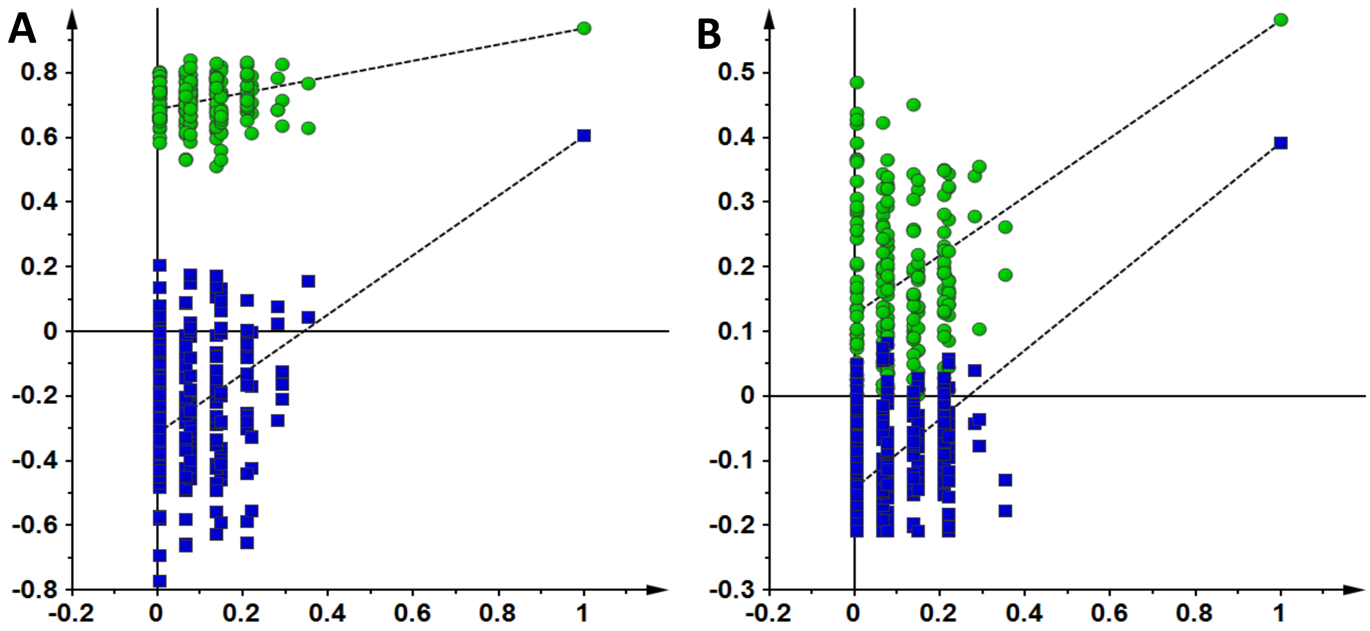

Figure S6. Permutation test showing the reliability of the separation obtained in the multidimensional model (PLS-DA) in advanced NSCLC stage. Panel A presents a permutation test of the PLS-DA model in advanced tissue samples (ESI+). Panel B presents a permutation test of the PLS-DA model in advanced tissue samples (ESI-).
